# Unraveling Circadian Rhythm Disorder-Related Gene Signatures and Molecular Subtypes in Ulcerative Colitis: An Analysis of Bulk and Single-Cell Transcriptomics

**DOI:** 10.3390/genes17040383

**Published:** 2026-03-27

**Authors:** Meng Sun, Xiaowei Fu, Xiaoyun Zhu, Dingqiao Xu, Shengyu Zhang, Yingshu Tan, Yaqing Mao, Yongming Li, Shanting Liao

**Affiliations:** 1 School of Medicine, Nanjing University of Chinese Medicine, Nanjing 210023, China; 17763278976@163.com (M.S.); vermissen12138@gmail.com (S.Z.); tanys0814@163.com (Y.T.); myq1325879@163.com (Y.M.); 2 School of Life Sciences, Westlake University, Hangzhou 310024, China; fuxiaowei@westlake.edu.cn; 3 Department of Pharmacology, Xiangya School of Pharmaceutical Sciences, Central South University, Changsha 410013, China; zhuxiaoyun@csu.edu.cn; 4 Hunan Provincial Key Laboratory of Cardiovascular Research, Central South University, Changsha 410013, China; 5 Key Laboratory of Shaanxi Administration of Traditional Chinese Medicine for TCM Compatibility, Shaanxi University of Chinese Medicine, Xianyang 712046, China; xudingqiao16@126.com

**Keywords:** circadian rhythm disorder, ulcerative colitis, gene signatures, molecular subtypes, single cell

## Abstract

Background: Ulcerative colitis (UC) is an intestinal disease characterized by long-term inflammation. Circadian rhythm disorder (CRD) affects various biological activities and has been linked to several diseases, including UC. This study aimed to investigate the role and significance of CRD in UC. Methods: Bulk RNA-seq data from five independent UC cohorts were obtained from the Gene Expression Omnibus (GEO) database and integrated into a single dataset. The dataset underwent differential analysis to identify differentially expressed genes (DEGs) in association with CRD. Expression levels and pathway enrichment of CRD genes were analyzed, and signature genes were identified using machine learning algorithms. Based on these signature genes, a UC risk prediction model and CRD-related molecular subtypes were established. Furthermore, single-cell RNA-seq data of UC were analyzed to discuss the key role of CRD and signature genes in the UC microenvironment. RT-PCR analysis was employed to validate the expression levels of the identified signature genes. Results: 247 DEGs associated with CRD in UC were identified (referred to as CRD-DEGs). Gene set enrichment analysis (GSEA) revealed a strong association between CRD and inflammation, as well as immune cell infiltration in UC. This association potentially impacts intestinal fibrosis. A comparison of three machine learning algorithms (Lasso, SVM-RFE, and Random Forest) resulted in the identification of 12 signature genes. A UC risk prediction model and two UC CRD subtypes were developed using these genes. Among them, STXBP1 was identified by all three machine learning algorithms and was further analyzed. STXBP1 was predominantly enriched in pathways related to inflammatory response. Elevated levels of STXBP1 are mainly caused by reduced levels of methylation of its gene promoter. RT-PCR confirmed elevated expression of certain genes in mouse UC models. Conclusions: This study is the first to establish a strong association between CRD and the onset of UC. The newly developed UC nomogram based on CRD demonstrated high predictive accuracy, although further clinical validation is required. Understanding the intrinsic relationship between CRD and UC enhances our understanding of the potential pathogenesis of UC. This study introduces novel ideas and methods for early diagnosis, treatment, and prognosis of UC.

## 1. Introduction

Ulcerative colitis UC is a chronic inflammatory disease that primarily affects the intestinal mucosa, resulting in inflammation confined to the mucosal surface [1]. The prevalence of UC has been increasing in recent years, and some patients even develop colorectal cancer [1]. Common clinical symptoms of UC include abdominal pain, bloody stools, and fecal urgency [2]. The pathogenesis of UC is multifactorial, involving environmental factors, genetic factors, and dysregulated immune responses [3,4]. Despite ongoing research, our understanding of the factors contributing to UC pathogenesis remains incomplete. Therefore, a comprehensive investigation of these factors can offer new insights into the treatment and prognosis.

Throughout evolution, mammals have developed an innate biological clock that responds to the light-dark cycles in their environment. Similarly, in healthy humans, the homeostatic system has adapted to these changes, resulting in circadian rhythms governing sleep, activity, and various physiological processes [5]. It has been observed that rhythmic variations also occur at the molecular level, involving substances like insulin and glucose [6,7]. Circadian rhythm disruptions have been associated with a range of diseases, and studies have indicated that individuals who work in shift schedules are more prone to developing diabetes, obesity, and cardiovascular disease. Sleep deprivation, for instance, leads to decreased levels of appetite-regulating hormones, potentially resulting in increased appetite, food intake, and a higher risk of obesity. Moreover, restricted sleep negatively affects glucose tolerance, insulin sensitivity, and the likelihood of developing diabetes [8,9].

Research conducted by Wang et al. has revealed that disruptions in circadian rhythms worsen colitis induced by dextran sulfate sodium (DSS) [10]. In another study, patients with inflammatory bowel disease (IBD) exhibit significantly lower expression levels of clock genes (CLOCK, CRY1, CRY2, PER1, and PER2) compared to healthy individuals. Additionally, patients with UC display greater leukopenia compared to those with Crohn’s disease [11]. These findings suggest a potential connection between circadian rhythm disturbances and colonic inflammation. However, the specific relationship and underlying mechanisms linking circadian rhythm disorders (CRD) and UC require further elucidation.

To elucidate the molecular mechanisms linking CRD and UC, we employed both bulk RNA sequencing and single-cell RNA sequencing approaches. Bulk transcriptomics provides averaged gene expression profiles across thousands of cells, offering high statistical power for detecting disease-associated gene expression changes and enabling the integration of large clinical cohorts. However, this approach masks cellular heterogeneity and rare cell population signals. Conversely, single-cell RNA sequencing (scRNA-seq) resolves transcriptional landscapes at individual cell resolution, enabling the identification of cell-type specific gene expression patterns and cellular crosstalk. The limitation of scRNA-seq includes higher technical noise, lower throughput for sample numbers, and substantially higher costs. By integrating both approaches, this study leverages the statistical robustness of bulk data for biomarker discovery and the cellular precision of scRNA-seq for mechanistic interpretation.

In summary, this study aimed to investigate the relationship between CRD and the pathogenesis of UC. To achieve this, we obtained bulk single-cell RNA-seq data from UC patients and normal tissues from the Gene Expression Omnibus (GEO) database. Differential analysis was performed to identify differentially expressed genes (DEGs) associated with CRD in the UC group, and these genes were further analyzed from multiple perspectives. Additionally, machine learning algorithms were utilized to identify 12 signature genes that were associated with CRD in UC. Using these signature genes, we developed new UC risk prediction models and CRD subtypes. Furthermore, we examined immune infiltration and pathway enrichment in different subtypes.

## 2. Methods

### 2.1. Data Collection and Processing

A comprehensive search was conducted in the GEO database to identify relevant bulk RNA-seq data pertaining to UC using the keyword “ulcerative colitis.” The dataset selection process involved rigorous inclusion and exclusion criteria. Inclusion criteria encompassed datasets with genome-wide expression profiles, specifically comparing healthy control (HC) samples with UC patients. Additionally, each dataset had to include a minimum of 10 UC samples and eight HC samples, with the specimens originating from human colon mucosal biopsy samples. Samples related to IBDs other than UC were excluded. To ensure reproducibility and transparency, the raw gene expression data for UC, comprising binary files in CEL format, were retrieved from the GEO database (https://www.ncbi.nlm.nih.gov/geo/) on 21 May 2023. Specifically, nine datasets (GSE87466, GSE75214, GSE206285, GSE87473, GSE47908, GSE16879, GSE48958, GSE53306, and GSE38713) were downloaded using the GEOquery package in R (version 4.2.1). These datasets were subsequently categorized into training and validation cohorts for further analysis, with detailed metadata summarized in Table 1.

To ensure comparability across the five independent training datasets (GSE87466, GSE75214, GSE206285, GSE87473, and GSE47908), we implemented a rigorous preprocessing pipeline [12]. First, raw CEL files were processed using the Robust Multi-array Average (RMA) method for background correction, quantile normalization, and summarization. Probe IDs were mapped to official gene symbols using the latest annotation files for each platform (GPL570, GPL6244, GPL13158). For genes represented by multiple probes, the expression value was calculated as the median of the corresponding probes. To address platform heterogeneity, we retained only the intersection of gene symbols common to all five datasets, resulting in a unified gene expression matrix. Subsequently, to remove non-biological batch effects arising from different studies, platforms, and processing times, we applied the ComBat algorithm from the Bioconductor sva package (version 3.46.0). The batch variable was defined as the GEO dataset ID (GSE number). Crucially, to preserve biological differences related to the disease, UC disease status (UC vs. HC) was included as a covariate in the model matrix during the ComBat adjustment. The final integrated training dataset comprised 879 UC samples and 86 healthy controls, with detailed sample composition listed in Table 1. The same preprocessing pipeline (RMA normalization, probe-to-gene mapping, gene intersection filtering, and ComBat batch correction) was applied to four additional datasets (GSE16879, GSE48958, GSE53306, and GSE38713) for validation purposes.

CRD-related genes were obtained by querying the GeneCards database using the keyword “circadian rhythm disorder” with a relevance score ≥ 5. In addition, circadian-related gene sets were retrieved from the Molecular Signatures Database (MSigDB), including KEGG_CIRCADIAN_RHYTHM_MAMMAL, REACTOME_BMAL1_CLOCK_NPAS2_ACTIVATES_CIRCADIAN_GENE_EXPRESSION, and WP_CIRCADIAN_RHYTHM_GENES. The genes from these sources were merged, and duplicate entries were removed to generate the final CRD-related gene list, comprising 2117 unique genes.

All data processing, integration, and batch correction were performed using R software (version 4.2.1). The major R packages used in this study and their versions are as follows: limma (3.54.2), glmnet (4.1-7), Seurat (4.3.0), clusterProfiler (4.6.2), Consensus-ClusterPlus (1.60.0), and GSVA (1.46.0).

### 2.2. Identification and Functional Enrichment of DEGs Associated with UC

Prior to differential expression analysis, a quality control step was performed to remove unreliable probes. The median expression values of less than 5.55 intensity on the log2 scale for each probe were filtered out, indicating the failure of true hybridization.

In the combined dataset, DEGs related to CRD (CRD-DEGs) were identified between UC and normal samples using the following threshold criteria: |log2 fold change (FC)| > 0.5 and adjusted *p*-value < 0.05. The adjusted *p*-values were calculated using the Benjamini–Hochberg (BH) method to control the false discovery rate (FDR) for multiple testing. The results were visualized using volcano plots and heatmaps generated with the “limma” R package [13]. Subsequently, Gene Ontology (GO) enrichment analysis and Kyoto Encyclopedia of Genes and Genomes (KEGG) pathway analysis were performed on the DEGs using the “clusterProfiler” package in R [14].

### 2.3. Immune Cell Infiltration Analysis

Considering the unique immune pathogenesis of UC, understanding immune cell infiltration is crucial for comprehending disease progression and treatment. Single-sample gene set enrichment analysis (ssGSEA), an extension of the gene set enrichment analysis (GSEA) method, was employed for assessing the relative abundance of infiltrating immune cells and the immunological signature in all UC and normal tissue samples. The “GSVA” package in R, based on the ssGSEA algorithm [15], was used for this analysis.

### 2.4. Identification of Signature Genes by Machine Learning

To identify potential biomarkers for UC, the CRD-DEGs were subjected to further screening using three machine learning algorithms: Least Absolute Shrinkage and Selection Operator (LASSO), Support Vector Machine Recursive Feature Elimination (SVM-RFE), and Random Forest (RF). LASSO regression was chosen for its ability to handle multicollinearity in high-dimensional data and produce sparse solutions via regularization. SVM-RFE is particularly effective for high-dimensional gene expression data with small sample sizes, as it recursively eliminates features to find the optimal subset. Random Forest (RF) was included to capture non-linear relationships and provide robust feature importance rankings. Employing all three algorithms allows for a consensus approach, enhancing the robustness of the identified biomarkers.

LASSO is a regularization method that introduces a penalty value (λ or shrinkage operator) to identify the best model. It applies a penalty function that shrinks the regression coefficients of variables, addressing covariance issues and preventing overfitting. In this study, the LASSO algorithm was implemented using the “glmnet” package in R. SVM-RFE, based on the “caret” package, assigns weights to variables using the Support Vector Machine algorithm. It iteratively selects smaller feature subsets through recursive screening and employs the RFE algorithm to determine the optimal subset [16]. The algorithm obtains the optimal variables through 10-fold cross-validation. The RF algorithm, based on the “Random Forest” package, predicts continuous variables and provides more stable predictions for accurate UC biomarkers.

To evaluate the predictive performance of the models comprehensively, we calculated Accuracy, Specificity, Sensitivity (Recall), and F1-score based on the confusion matrix (TP: True Positive, TN: True Negative, FP: False Positive, FN: False Negative). The equations are defined as follows:

Accuracy = (TP + TN)/(TP + TN + FP + FN)

Sensitivity (Recall) = TP/(TP + FN)

Specificity = TN/(TN + FP)

F1-score = 2 × (Precision × Recall)/(Precision + Recall) = 2TP/(2TP + FP + FN)

where Precision = TP/(TP + FP).

Subsequently, the predictive performance of the feature genes obtained from the three machine learning algorithms was evaluated using the validation set. The results were visualized as receiver operating characteristic (ROC) curves, and the cumulative residual distribution of the three models was plotted to compare their screening performance.

### 2.5. Construction and Validation of the Predictive Model

The UC risk prediction model, based on the identified signature genes, was constructed using the “ggplot2” package to build a nomogram. ROC analysis was performed using the “pROC” R package to assess the predictive performance of the model. To verify the accuracy of the nomogram, a calibration curve was generated using the “pacman” package. The clinical predictive value of the model was assessed through decision curve analysis (DCA) using the “limma” package in R. Additionally, the predictive performance of the model was evaluated in four independent test cohorts using ROC analysis.

### 2.6. Identification of CRD Subtypes in UC

Unsupervised hierarchical clustering analysis was employed to classify the 879 UC tissue samples into different clusters based on the 12 hallmark genes identified by the machine learning models [17]. To determine the optimal number of clusters, principal component analysis (PCA), cumulative distribution function (CDF) curves, and consensus cluster scores were utilized. Gene Set Variation Analysis (GSVA) was performed on UC pathways within the identified subclusters of CRD using cluster analysis. Heatmaps were generated to visualize the results. The immune microenvironment of the two different clusters was evaluated to compare the differences in immune cell infiltration. The intersection of genes from the three machine learning models was selected for further analysis. Differential expression analysis was conducted between high and low levels of STXBP1 in UC patients, and functional enrichment analysis was performed on the DEGs to describe their biological functions. These analyses were carried out using relevant R packages, including “ConsensusClusterPlus,” “limma,” “ClusterProfiler,” “GSVA,” and “GSEABase.” Statistical significance was determined based on |log2FC| > 0.5 and adjusted *p*-value < 0.05.

### 2.7. Data Processing of 10× scRNA-Seq

The 10× scRNA-Seq data from UC patients (GSE231993) were downloaded from the GEO database. This dataset includes colonic biopsy samples from 4 UC patients and 4 HC. Raw gene expression matrices generated using the 10× Genomics platform were downloaded, and all downstream analyses were performed independently in this study.

Data preprocessing and analysis were conducted using the Seurat R package. Quality control (QC) filtering was applied to remove low-quality cells based on the following criteria: cells with gene counts between 200 and 5000 were retained, and cells with mitochondrial gene percentage exceeding 20% were excluded to eliminate low-quality or stressed cells. After QC, the data were normalized using the “LogNormalize” method with a scale factor of 10,000. The top 2000 highly variable genes were identified using the “FindVariableFeatures” function with the “vst” selection method. PCA was performed using the top 2000 variable genes, and the first 20 principal components (PCs) were selected for downstream dimensionality reduction and clustering based on the elbow plot of PC standard deviations.

For cell clustering, the “FindClusters” function was applied with a resolution parameter of 0.5, which was optimized to achieve stable and biologically meaningful cluster separation. Dimensionality reduction and visualization were performed using uniform manifold approximation and projection (UMAP) and t-distributed stochastic neighbor embedding (t-SNE). Cell type annotation was performed using a combined strategy of automated annotation and manual curation. Automated annotation was conducted using the “SingleR” R package with the Human Primary Cell Atlas as the reference dataset. Manual curation was performed based on the expression of canonical marker genes, including B cells (CD79A, CD37), T cells (CD3D, CD3E), epithelial cells (CDH1, CLDN4), smooth muscle cells (ACTA2, TAGLN), Monocyte (CD14, FCN1), endothelial cells (PECAM1, VWF), fibroblasts (FGF7, MME), neurons (ENO2), and macrophages (CD68, CD163).

### 2.8. Data Processing of DNA Methylation

The DNA methylation dataset of UC (GSE81211) included normal colon samples from HCs and colon samples from active UC patients. The dataset was obtained from the GEO database. Methylation profiling was performed using the Illumina Infinium 450k Human DNA methylation platform, which encompasses more than 480,000 CpGs. Promoters were categorized into high-CpG promoters (HCP), intermediate CpG promoters (ICP), and low-CpG promoters (LCP) based on CpG ratio, CpG content, and CpG region length. The methylation level of a gene promoter region was determined by calculating the average methylation level across all CpG sites within the region. Differentially methylated promoters (DMPs) were identified as promoters exhibiting the highest and lowest 5% differential methylation between UC patients and HCs, as determined by *t*-test rankings.

### 2.9. CRD Model

Male C57BL/6J mice (4 weeks old) were purchased from GemPharmatech Co., Ltd. (Nanjing, China). Mice were all free of specific pathogens and were kept under controlled environmental conditions (21–25 °C, 50–60% humidity) with ad libitum access to food and water.

To induce circadian rhythm disruption, mice were subjected to a light-dark phase shift protocol as previously described with minor modifications [18]. Briefly, mice were housed in light-controlled chambers. Control mice were maintained under a standard 12 h light/12 h dark cycle (lights on at 08:00 and lights off at 20:00). For the CRD mice, the light-dark cycle was delayed by 6 h each week, resulting in a progressive shift in the photoperiod. Specifically, the light onset was adjusted from 08:00–20:00 in week 1 to 14:00–02:00 in week 2, 20:00–08:00 in week 3, and 02:00–14:00 in week 4. This phase-delay shifting schedule was maintained throughout the experimental period to induce chronic circadian misalignment.

### 2.10. DSS-Induced Colitis Model

C57BL/6 mice were assigned to two groups (*n* = 4 per group): the control group and the CRD mice induced colitis by DSS (DSS group). To induce colitis, CRD mice were provided with 2.5% (*w*/*v*) DSS in their drinking water for 7 consecutive days. Control mice had free access to food and water. Following this period, all mice were euthanized via isoflurane anesthesia. The colons were then excised and immediately frozen in liquid nitrogen for subsequent total RNA extraction.

All animal experiments were approved by the University Committee on Use and Care of Animals of Nanjing University of Chinese Medicine.

### 2.11. Reverse Transcription-Quantitative PCR

Total RNA was extracted using RNA isolater Total RNA Extraction Reagent (Vazyme, Nanjing, China), and cDNA synthesis was performed using Hifair^®^ III 1st Strand cDNA Synthesis SuperMix for qPCR (gDNA digester plus) (Yeasen, Shanghai, China). Reverse transcription-quantitative PCR was performed with Hieff^®^ qPCR SYBR Green Master Mix (Low Rox Plus) (Yeasen, Shanghai, China) on the QuantStudio 3 Real-Time PCR System (Thermo Fisher Scientific, Waltham, MA, USA). Relative mRNA expression levels were standardized on the β-Actin gene. The sequences of primer sets designed to detect specific genes are listed in Table 2.

### 2.12. Statistical Analysis

All data processing, statistical analysis, and plotting were carried out using R software. For continuous variables (e.g., gene expression levels derived from RNA-seq and single-cell RNA-seq, methylation beta values, and immune cell infiltration scores), data normality was first assessed. Differences between two groups (UC vs. HC) were evaluated using an unpaired two-tailed Student’s *t*-test for normally distributed data, or the Wilcoxon rank-sum test for non-normally distributed data. For categorical variables (e.g., gender, disease stage, or other clinical characteristics), group differences were assessed using the chi-square (χ^2^) test. When the expected frequency in any cell of the contingency table was less than 5, Fisher’s exact test was applied instead.

Reverse transcription-quantitative PCR data are presented as mean ± SD. Statistical analysis was performed using GraphPad Prism 10.1.2 (GraphPad Software, Boston, MA, USA). Differences between groups were analyzed using an unpaired two-tailed Student’s *t*-test.

For high-throughput omics analyses, including differential expression analysis, functional enrichment, and immune infiltration comparisons, statistical significance was defined by an adjusted *p*-value < 0.05 using the BH method to control the FDR. For Reverse transcription-quantitative PCR data, statistical significance was defined by a raw *p*-value < 0.05.

## 3. Results

### 3.1. Differential Gene Screening and Functional and Pathway Enrichment Analysis

**Figure 1:**
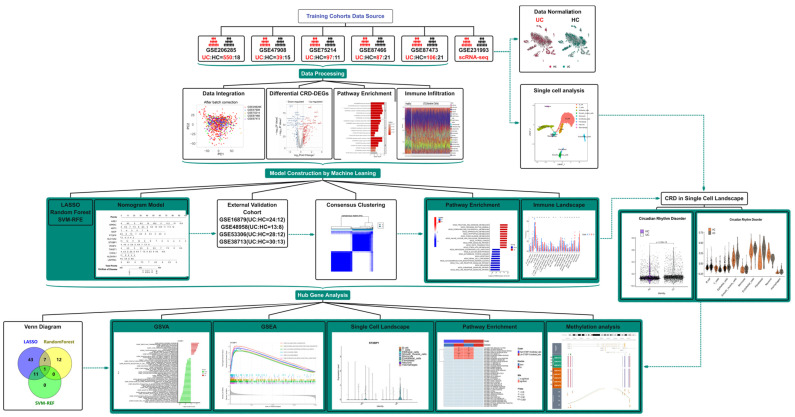
The flow chart that summarizes this study.

**Figure 2:**
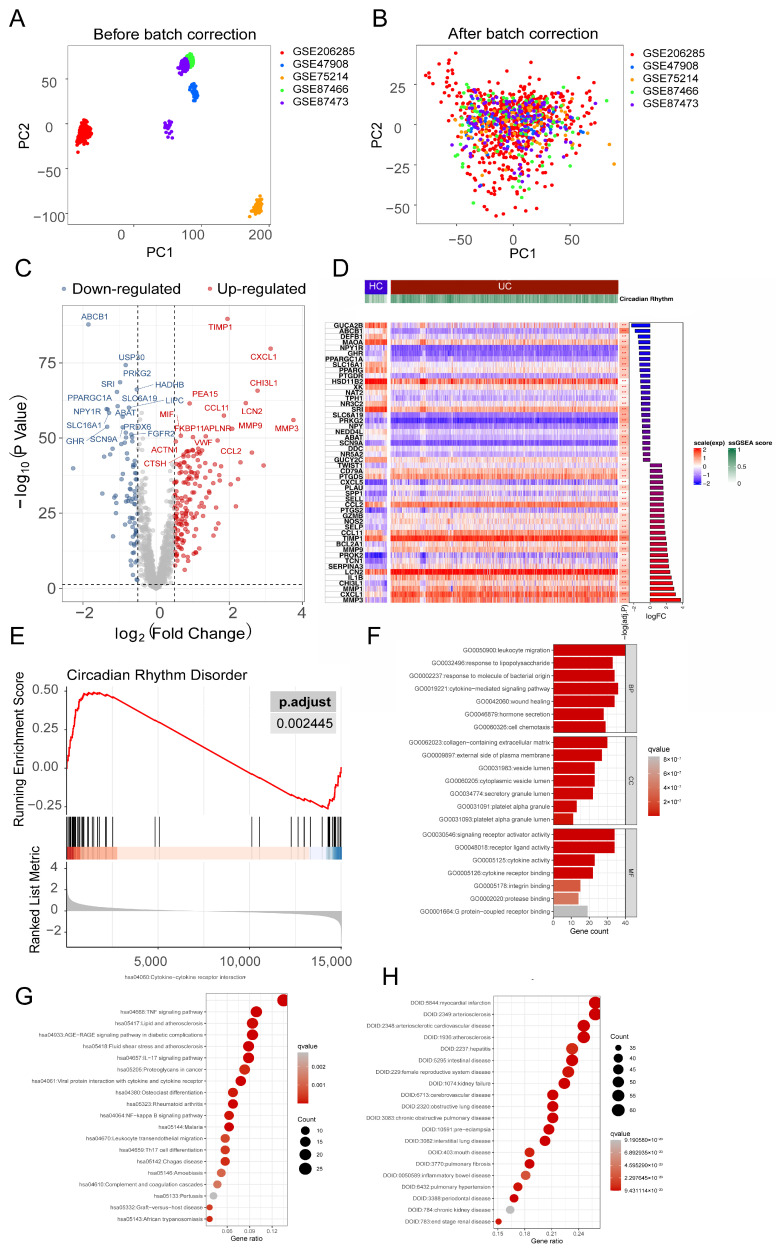
Identification and functional enrichment of CRD-DEGs in UC. (**A**,**B**) PCA plots of five integrated GEO datasets (GSE206285, GSE47908, GSE75214, GSE87466, GSE87473) before (**A**) and after (**B**) batch effect correction. (**C**) Volcano plot showing CRD-DEGs between UC (*n* = 879) and HC, *n* = 86. (i) Non-significant genes (grey dots): |log2FC| ≤ 0.5 or adjusted *p*-value ≥ 0.05; (ii) Upregulated genes (red dots): log2FC > 0.5 and adjusted *p*-value < 0.05; (iii) Downregulated genes (blue dots): log2FC < −0.5 and adjusted *p*-value < 0.05. The horizontal dashed line indicates adjusted *p*-value = 0.05 (−log10 scale); vertical dashed lines indicate log2FC = ±0.5. (**D**) Heatmap of CRD-DEGs. Rows represent genes, columns represent samples. (**E**) GSEA results of CRD-DEGs. (**F**–**H**) Functional enrichment analyses of CRD-DEGs: (**F**) GO, (**G**) KEGG, and (**H**) DO.

Figure 1 shows the workflow of this study. We conducted PCA and applied normalization and batch correction (Figure 2A,B and Figure S1A,B) on five datasets (GSE206285, GSE47908, GSE75214, GSE87466, and GSE87473) to generate a combined dataset comprising 86 normal colon tissues and 879 UC tissue samples. Subsequently, differential analysis was conducted on the combined dataset. The volcano plot displays CRD-DEGs. In comparison to the normal group, the UC group exhibited 162 up-regulated CRD-DEGs and 85 down-regulated CRD-DEGs (Figure 2C). Additionally, the top 20 up- and down-regulated genes were depicted in the heatmap (Figure 2D), and the ssGSEA score was higher in the UC group. To further explore the association with CRD, we established a gene set specifically related to CRD and performed a GSEA (Figure 2E), which indicated significant enrichment of genes associated with CRD in the UC group. Subsequently, we conducted Gene Ontology (GO) enrichment analysis (Figure 2F), Kyoto Encyclopedia of Genes and Genomes (KEGG) pathway enrichment analysis (Figure 2G), and Disease Ontology (DO) enrichment analysis (Figure 2H) on the identified CRD-DEGs. The results revealed that these CRD-DEGs were involved in various signaling pathways, including cytokine-cytokine receptor interaction, cellular chemotaxis, and IL-17 signaling. They also participated in biological activities such as leukocyte migration, LPS response, cell differentiation, and immune response. Notably, the DO analysis demonstrated a clear association between these CRD-DEGs and intestinal diseases, highlighting their relevance in this context.

### 3.2. Analysis of Immune Cell Infiltration in UC Patients

**Figure 3:**
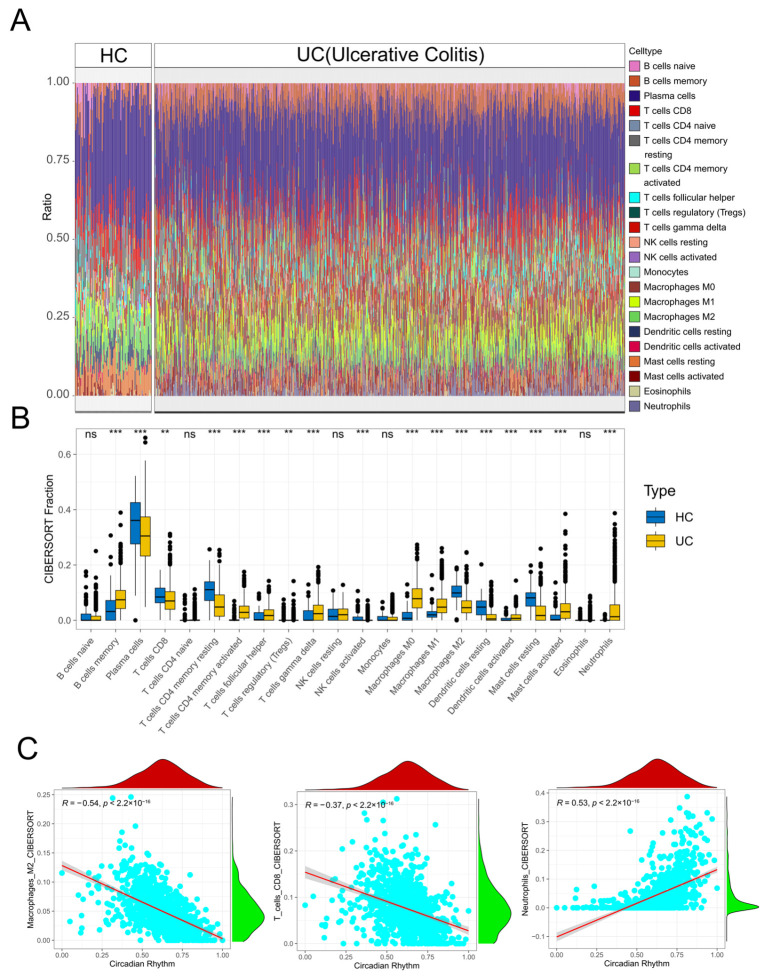
Immune cell infiltration landscape in UC and its correlation with CRD-DEGs. (**A**) Heatmap showing the relative abundance of 22 immune cell types in UC and HC samples. (**B**) Box plots comparing immune cell infiltration levels between UC and HC groups. ** *p* < 0.01, *** *p* < 0.001; ns, not significant. (**C**) Scatter plot of the correlation between CRD-DEGs and immune cell infiltration.

To further investigate the impact of CRD on immune cells in UC patients, we employed the CIBERSORT algorithm in the enrichment analysis to assess the composition of immune cells in different samples (Figure 3A,B). The results indicated significant differences in immune cell composition between UC patients and HCs. In comparison to the normal group, UC patients exhibited elevated levels of memory B cells, activated memory CD4 T cells, M0 macrophages, M1 macrophages, activated mast cells, and neutrophils. Conversely, they displayed reduced levels of resting memory CD4 T cells, CD8 T cells, M2 macrophages, and resting mast cells. These findings underscored a higher degree of inflammation in UC patients compared to the normal group, which aligned with expectations.

Next, we examined the correlation between CRD and immune cell infiltration in UC patients (Figure 3C). Notably, we observed a negative correlation between CRD and the infiltration of M2 macrophages and CD8 T cells, indicating that as CRD increased, the levels of these cell types decreased. Conversely, there was a positive correlation between CRD and the infiltration of neutrophils, suggesting that their levels gradually increased with CRD. This strong correlation between CRD and immune cell infiltration was further supported by the Supplementary Data (Figure S1).

### 3.3. Effects of CRD on Cells in the Colonic Region

**Figure 4:**
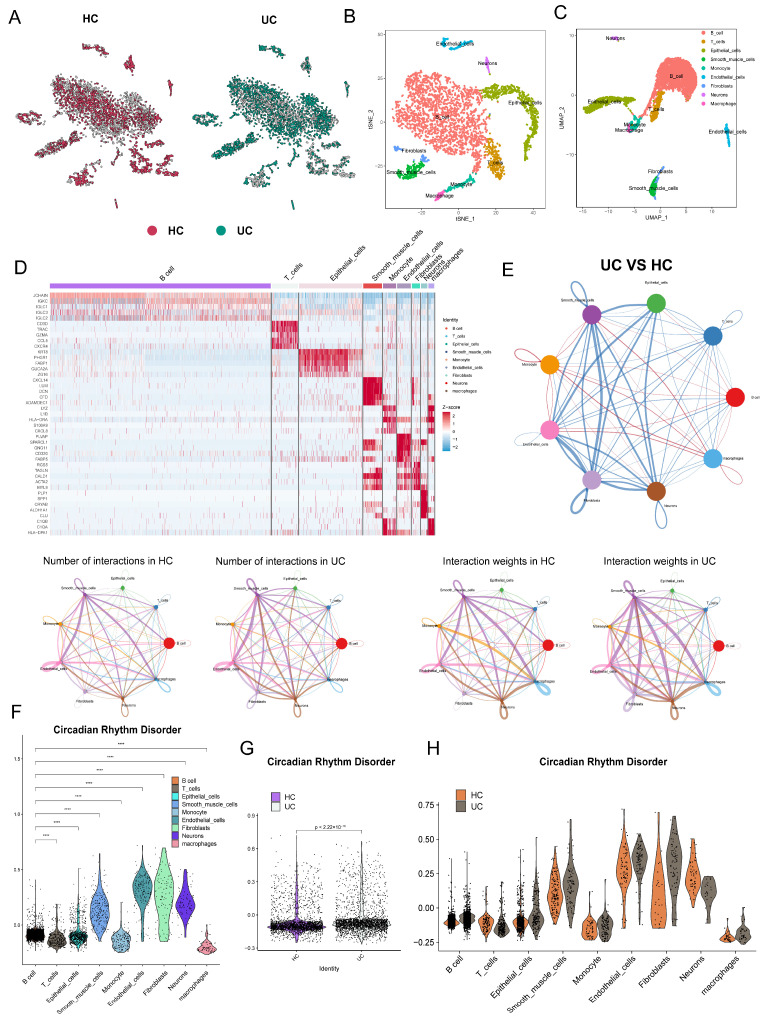
Single-cell transcriptomic landscape of UC and CRD-related alterations. (**A**) t-SNE plots of cells from normal and UC samples after integration and batch correction. (**B**,**C**) Dimensionality reduction visualization of all cells by t-SNE (**B**) and UMAP (**C**), with cells colored by cell type annotation based on marker genes. (**D**) Heatmap showing the top marker genes for each major cell type. (**E**) Intercellular communication network showing the number (left) and strength (right) of ligand-receptor interactions among cell types. (**F**–**H**) Comparison of CRD-related gene set expression across cell types (**F**), overall between groups (**G**), and between UC and HC groups within the same cell type (**H**). **** *p* < 0.0001.

To gain further insight into the effects of CRD on cells in the colonic region of UC patients, we analyzed scRNA-seq data obtained from both UC and normal tissues. After segregating the cells into the HC and UC groups (Figure 4A), we employed the UMAP and t-distributed stochastic neighborhood embedding (t-SNE) dimensionality reduction algorithms on this dataset. The results are visualized in Figure 4B, with nine labeled cell types. The heatmap (Figure 4C) illustrates the genes that were enriched in these distinct cell types.

Next, we investigated the number and strength of interactions between these cells (Figure 4D,E). Notably, there was a significant increase in the interactions between fibroblasts, smooth muscle cells, and neurons in the UC group. This observation highlighted the enhanced intercellular communication and crosstalk among these specific cell types in UC patients. By integrating the expression of relevant CRD indicators in the aforementioned cells, we generated Figure 4F–H. Through comparing the expression of CRD indicators across different cells, different groups within the same cell type, and different groups collectively, we observed a significant upregulation of CRD-related indicators in B cells, smooth muscle cells, endothelial cells, and fibroblasts. This finding suggested a potential involvement of these cell types in the dysregulation of circadian rhythm in UC patients.

### 3.4. Screening for Signature Genes Associated with CRD in UC by Machine Learning Algorithms

**Figure 5:**
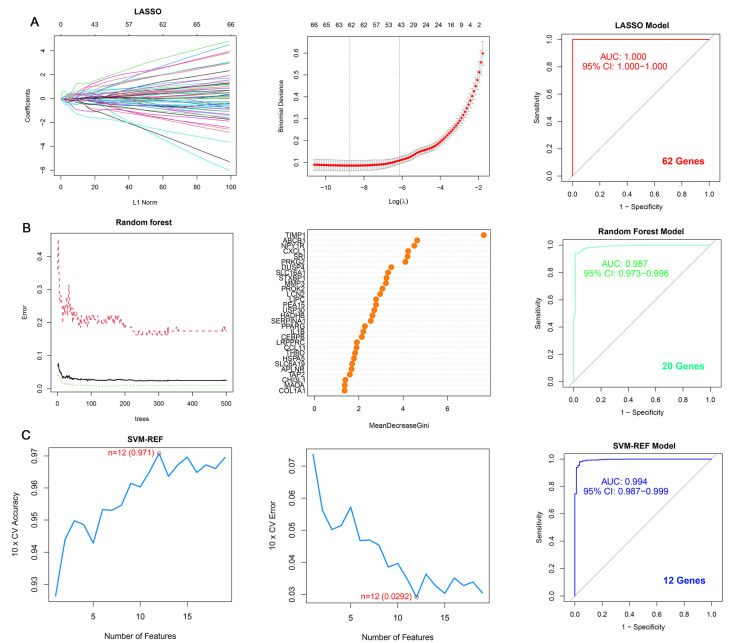
Identification of signature genes by machine learning algorithms. (**A**) LASSO regression analysis. (**B**) RF algorithm. The top 20 genes with relative importance > 2 were selected as candidate signature genes. (**C**) SVM-RFE algorithm. The classifier achieved minimal error with 12 features. ROC curves and AUC values for each algorithm are shown on the right panels, demonstrating the predictive performance in the training set.

In order to further refine the characterization of the results, we employed machine learning algorithms to screen the genes associated with CRD in UC (Figure 5). Using three algorithms, namely LASSO regression, RF, and SVM-RFE, we obtained three sets of signature genes and evaluated the performance of the algorithms through ROC curves, area under the curve (AUC) values, and the number of signature genes. Applying the LASSO algorithm, we identified 62 potential signature genes that displayed significant relevance to CRD in UC. For the RF algorithm, we identified 20 genes with relative importance greater than 2, indicating their significance as signature genes. As for the SVM-RFE algorithm, the classifier achieved the minimum error when the number of features was 12, leading us to identify 12 valid signature genes.

To comprehensively evaluate the predictive performance of the three algorithms, we calculated Accuracy, Sensitivity (Recall), Specificity, and F1-score on the validation set. As shown in Table 3, LASSO achieved the highest accuracy (0.985) and a perfect AUC of 1.000, but identified 62 signature genes. RF identified 20 signature genes with an AUC of 0.987 and an accuracy of 0.942. SVM-RFE achieved a near-perfect AUC of 0.994 (95% CI: 0.987–0.999) and an accuracy of 0.968, while identifying only 12 signature genes. Although the LASSO model showed a perfect AUC of 1.000 in the validation cohort, it identified 62 signature genes. In contrast, the SVM-RFE model achieved a comparably excellent AUC of 0.994 with only 12 genes. A model with fewer biomarkers (12 vs. 62) is more cost-effective and easier to translate into clinical diagnostic applications. Additionally, the SVM-RFE model demonstrated high internal stability with a 10-fold cross-validation accuracy of 0.971 and a low error rate of 0.0292 at the optimal feature count (*n* = 12). An AUC of exactly 1.000 (as seen in LASSO) can sometimes indicate overfitting to the specific noise of the dataset, whereas the slightly lower but still near-perfect AUC of SVM-RFE, combined with rigorous cross-validation, suggests a more robust generalization capability for broader populations. Therefore, considering the balance between predictive performance, model complexity, and clinical applicability, we selected the SVM-RFE algorithm with 12 signature genes as the optimal model.

**Figure 6:**
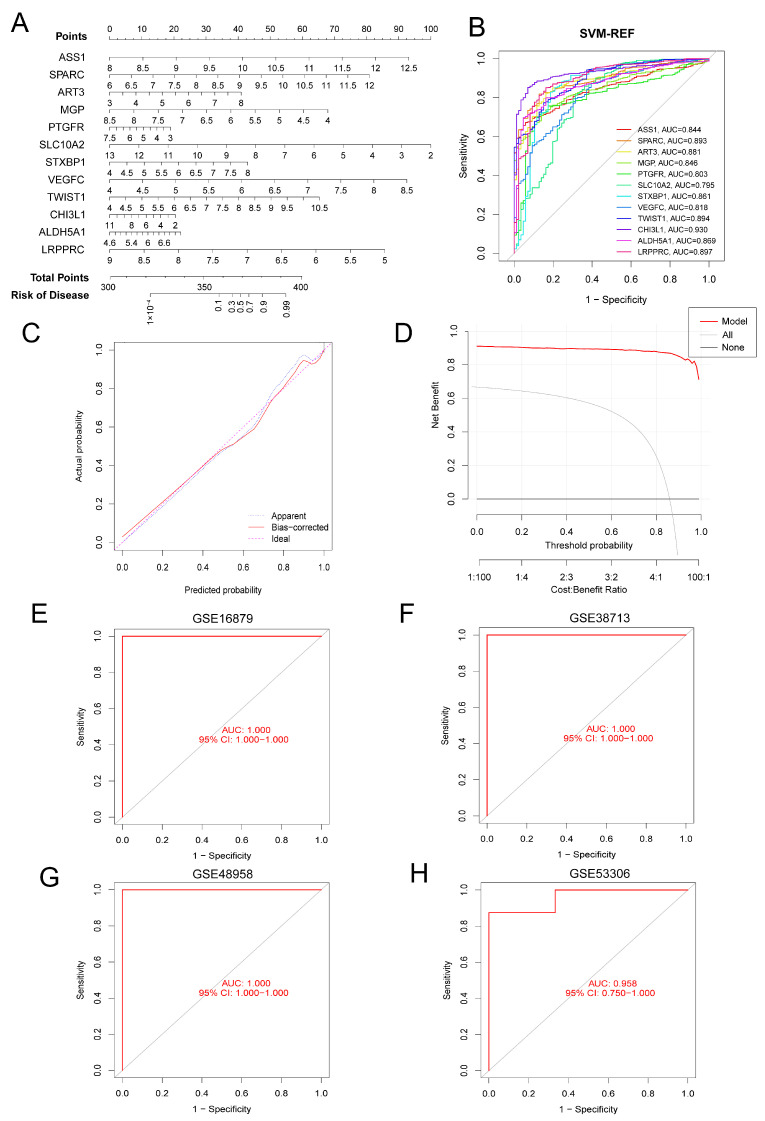
Construction and validation of a UC risk prediction nomogram based on 12 signature genes. (**A**) Nomogram for predicting UC risk. (**B**) ROC curves of the 12 signature genes individually in the training set. AUC values are shown in the legend. (**C**) Calibration curve of the nomogram, showing good agreement between predicted and observed probabilities. (**D**) Decision curves of the clinical values of the nomogram. (**E**–**H**) Validation of the nomogram in four independent test cohorts: (**E**) GSE16879, (**F**) GSE38713, (**G**) GSE48958, and (**H**) GSE53306. ROC curves and AUC values confirm robust predictive performance.

To enable early prediction and diagnosis of UC, we constructed a nomogram based on the 12 identified signature genes (Figure 6A). Each signature gene was assigned a corresponding score, and the total score was calculated by summing the scores of all signature genes. Different total scores corresponded to different levels of UC risk. The ROC curve (Figure 6B) demonstrated the excellent predictive value of each individual signature gene in distinguishing UC. We also included the ROC curves of predicted genes from the LASSO and RF algorithms for comparison (Figure S2). The calibration curve and clinical decision curve further confirmed the accuracy and clinical benefit of the nomogram (Figure 6C,D).

Moreover, in four independent test cohorts consisting of UC and HC samples (GSE16879, GSE38713, GSE48958, and GSE53306) (Figure 6E–H), the set of 12 signature genes obtained through the SVM-RFE algorithm exhibited excellent AUC values (indicating high predictive accuracy), which were 1.0, 1.0, 1.0, and 0.958, respectively. This further validated the ability of these signature genes to effectively distinguish between the normal and UC groups.

**Figure 7:**
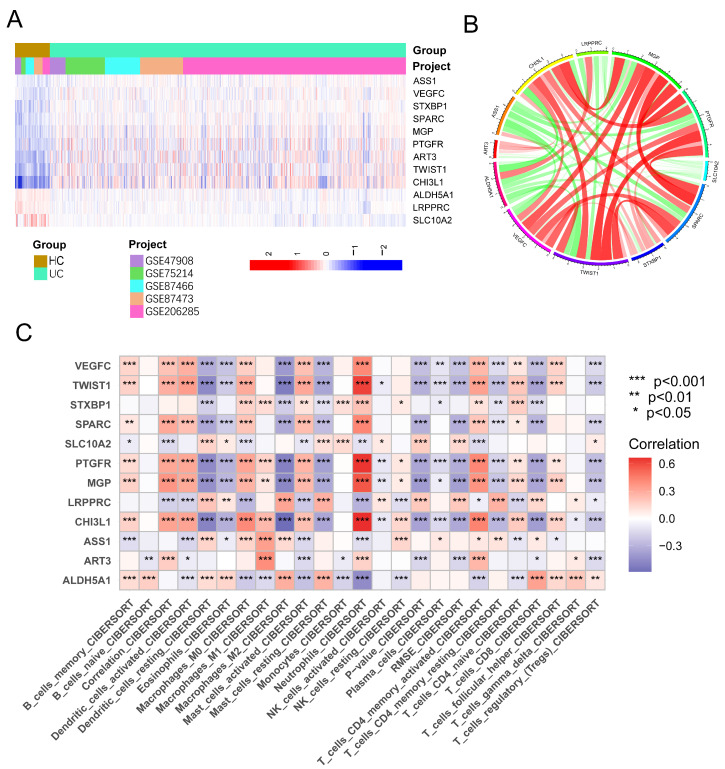
Expression patterns and immune correlations of the 12 signature genes. (**A**) Heatmap showing the expression levels of the 12 signature genes in UC and HC samples. Rows represent genes, columns represent samples. (**B**) Chord diagram illustrating the pairwise correlations among the 12 signature genes. (**C**) Heatmap showing Spearman correlations between the 12 signature genes and immune cell infiltration levels. Red: positive correlation; blue: negative correlation.

We presented the expression patterns of these 12 genes in the UC and HC groups using a heatmap (Figure 7A). It was evident that ASS1, VEGFC, STXBP1, SPARC, MGP, PTGFR, ART3, TWIST1, and CHI3L1 were up-regulated, while ALDH5A1, LRPPRC, and SLC10A2 were down-regulated in UC. The heatmap (Figure 7B) also revealed correlations among the 12 genes. Furthermore, the heatmap depicting the correlations between signature genes and immune cells demonstrated varying degrees of correlation between all 12 signature genes and immune cells. Notably, TWIST1, SPARC, PTGFR, MGP, and CHI3L1 exhibited a strong positive correlation with neutrophils and a strong negative correlation with M2 macrophages (Figure 7C).

### 3.5. Single-Cell Sequencing of Signature Genes

**Figure 8:**
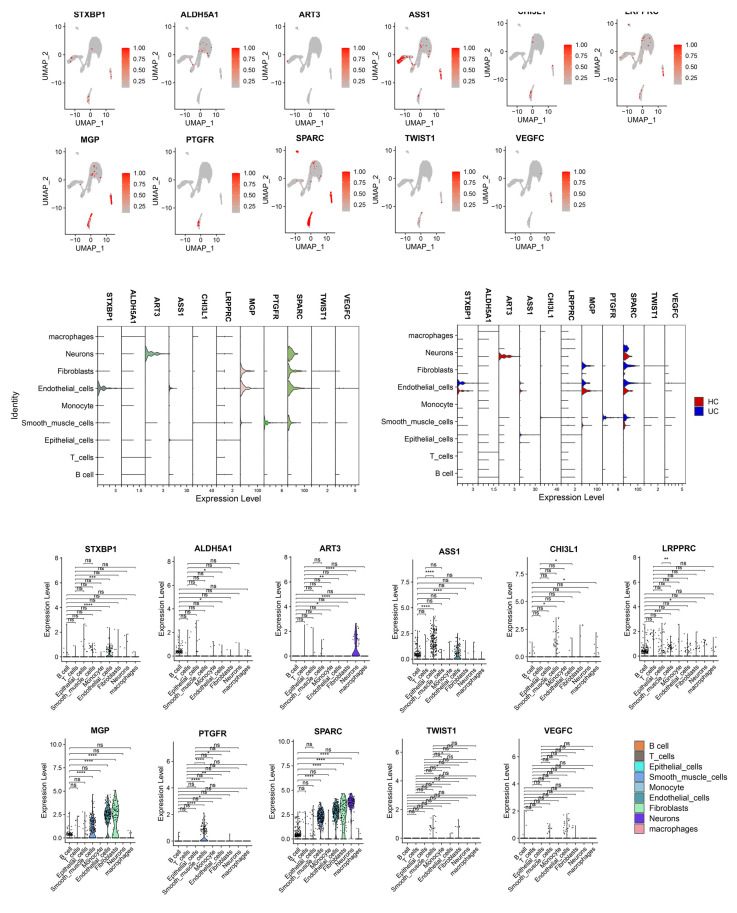
Single-cell expression patterns of the 12 signature genes. * *p* < 0.05, ** *p* < 0.01, *** *p* < 0.001, **** *p* < 0.0001; ns, not significant.

We reanalyzed the scRNA-seq data from Figure 4C to examine the expression of the signature genes across different cell types (Figure 8). This visualization allowed us to observe the expression patterns of different genes in specific cell populations.

STXBP1, ASS1, MGP, and SPARC exhibited predominant expression in endothelial cells, with STXBP1 showing upregulation in the UC group. ART3 and SPARC were primarily expressed in neurons, and ART3 displayed significant downregulation in the UC group. MGP and SPARC showed predominant expression in fibroblasts, and both genes were significantly up-regulated in the UC group. Furthermore, PTGFR and SPARC were predominantly expressed in smooth muscle cells, and they were significantly up-regulated in the UC group. These findings provided insights into the specific cell types associated with the dysregulation of these signature genes in UC.

### 3.6. Construction of UC CRD Subtypes

**Figure 9:**
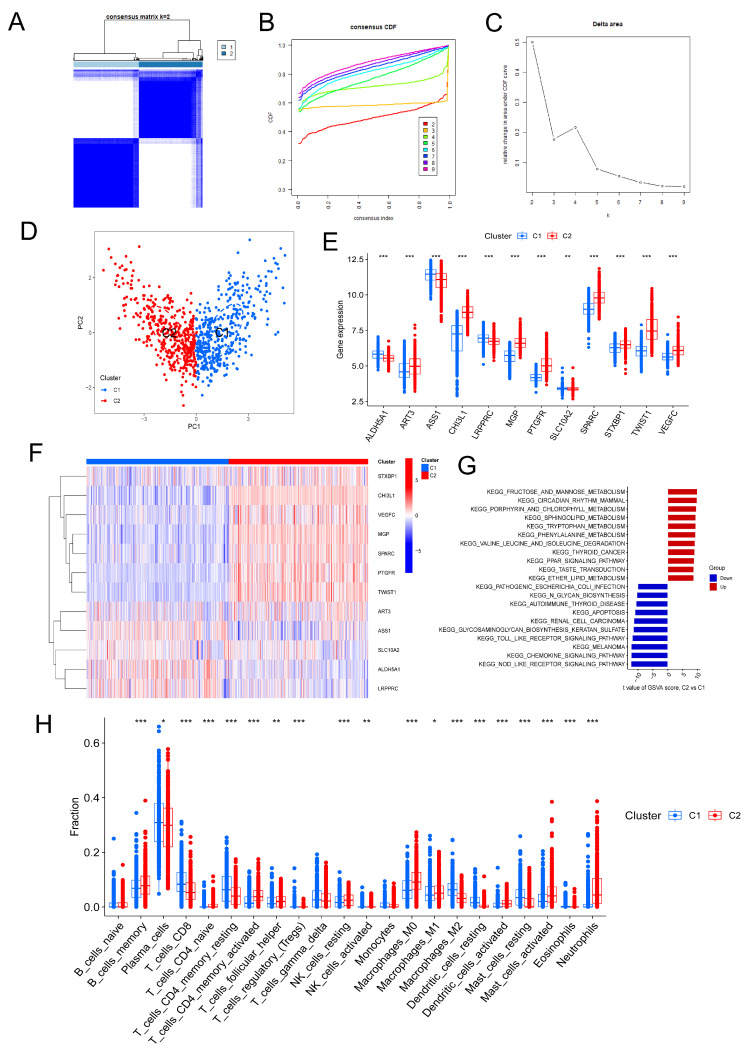
Identification of CRD-related molecular subtypes in UC. (**A**) Consensus clustering matrix when k = 2. (**B**) Consensus CDF delta area curves when k = 2–9. (**C**) Relative alterations in the AUC of CDF curve. (**D**) Principal component plots of the two subtype clusters. (**E**,**F**) Box plots and heatmaps of the expression of 12 genes in the two isoforms, with each row of the heatmap representing one gene and each column representing one sample. (**G**) GSVA of KEGG pathways in the two subtypes. (**H**) Box plots of the differences in immune cell infiltration in the two clusters. * *p* < 0.05, ** *p* < 0.01, *** *p* < 0.001.

Using the 12 signature genes, we conducted a consensus clustering analysis on the 879 UC samples. By examining the relative changes in consensus matrix plots, CDF curves, and AUC of CDF curves, we determined that the optimal grouping was achieved at k = 2 (Figure 9A–C). Consequently, we identified two CRD subtypes in UC: Cluster 1 and Cluster 2. PCA of these two subtype clusters (Figure 9D) demonstrated a clear separation between them.

The expression of the 12 genes in both subtypes is depicted in boxplots and heatmaps (Figure 9E,F). It was evident that the expression of ART3, CHI3L1, MGP, PTGFR, SPARC, STXBP1, TWIST1, and VEGFC was significantly higher in Cluster 2 compared to Cluster 1, while the opposite was true for ALDH5A1, ASS1, and LRPPRC. Furthermore, upon comparing the differences in immune cell infiltration between the two clusters (Figure 9H), we observed significantly higher levels of neutrophils, activated mast cells, M0 macrophages, activated memory CD4 T cells, and memory B cells in Cluster 2, whereas M2 macrophages, resting memory CD4 T cells, and CD8 T cells were significantly lower. These findings suggested that Cluster 2 was associated with more severe inflammation. To further investigate the differences in signaling pathways between the two UC subtype clusters, we conducted GSVA (Figure 9G). Compared to Cluster 1, Cluster 2 exhibited significant downregulation of the Toll-like receptor transduction pathway, chemokine signaling pathway, and NOD-like receptor signaling pathway. Additionally, Cluster 2 showed increased degradation levels of valine, leucine, and isoleucine, as well as elevated metabolic levels of phenylalanine, tryptophan, sphingolipids, porphyrin, chlorophyll, fructose, and mannose. These findings shed light on the distinct signaling pathway profiles associated with the two UC subtype clusters.

### 3.7. In-Depth Exploration of STXBP1

**Figure 10:**
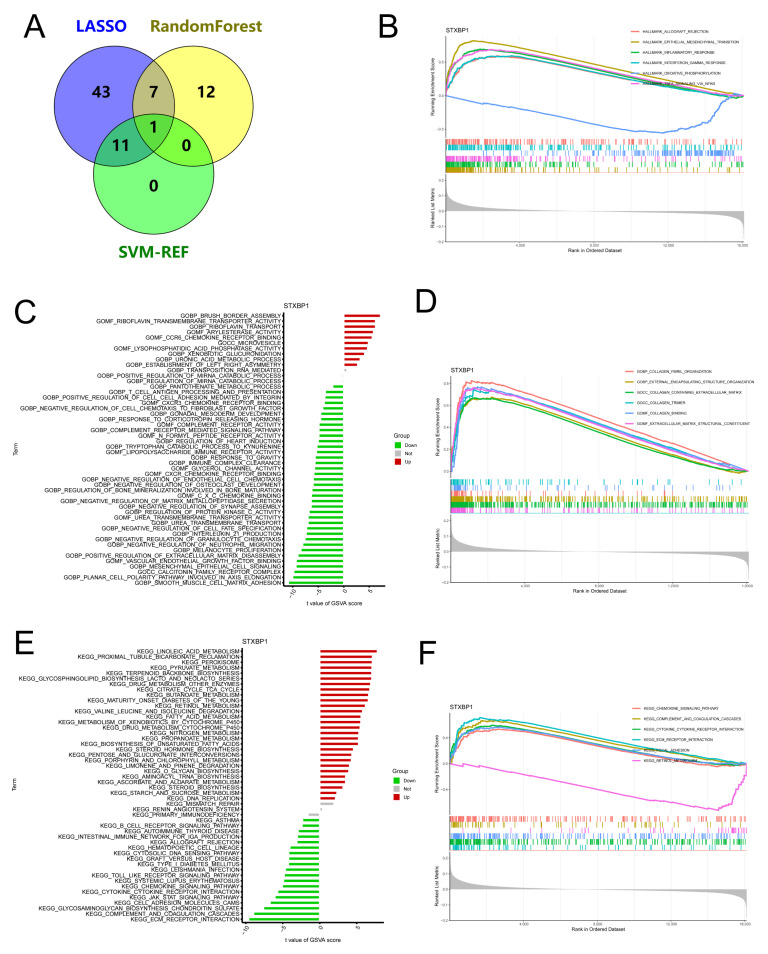
Functional characterization of STXBP1 in UC. (**A**) Venn diagram showing the intersection of signature genes identified by LASSO, RF, and SVM-RFE algorithms. (**B**,**D**,**F**) GSEA comparing the STXBP1 high-expression group versus low-expression group (grouped by median expression) using (**B**) Hallmark gene sets, (**D**) GO, and (**F**) KEGG pathways. (**C**,**E**) GSVA of (**C**) GO and (**E**) KEGG pathways between STXBP1 high- and low-expression groups.

To further investigate the function of STXBP1 in UC, we generated a Venn diagram of the signature genes obtained from the three previous algorithms (Figure 10A), revealing that STXBP1 was the gene common to all three algorithms. We subsequently divided the UC group into two subgroups based on STXBP1 expression: the STXBP1 high-expression group and the STXBP1 low-expression group. We performed GSEA on Hallmark, KEGG, and GO pathways to explore the functional implications of STXBP1 (Figure 10B,D,F). Notably, we observed significant enrichment of inflammation-related pathways in the STXBP1 high-expression group. Furthermore, we conducted GSVA on the DEGs between the two groups (Figure 10C,E). The analysis revealed several prominent pathways that were enriched in the STXBP1 high-expression group. These pathways included the reduction in smooth muscle cell–matrix adhesion, negative effects on vascular endothelial cells, inhibition of cytokine and cell adhesion molecule signaling, as well as enhancement of metabolic processes related to specific amino acids and salts.

**Figure 11:**
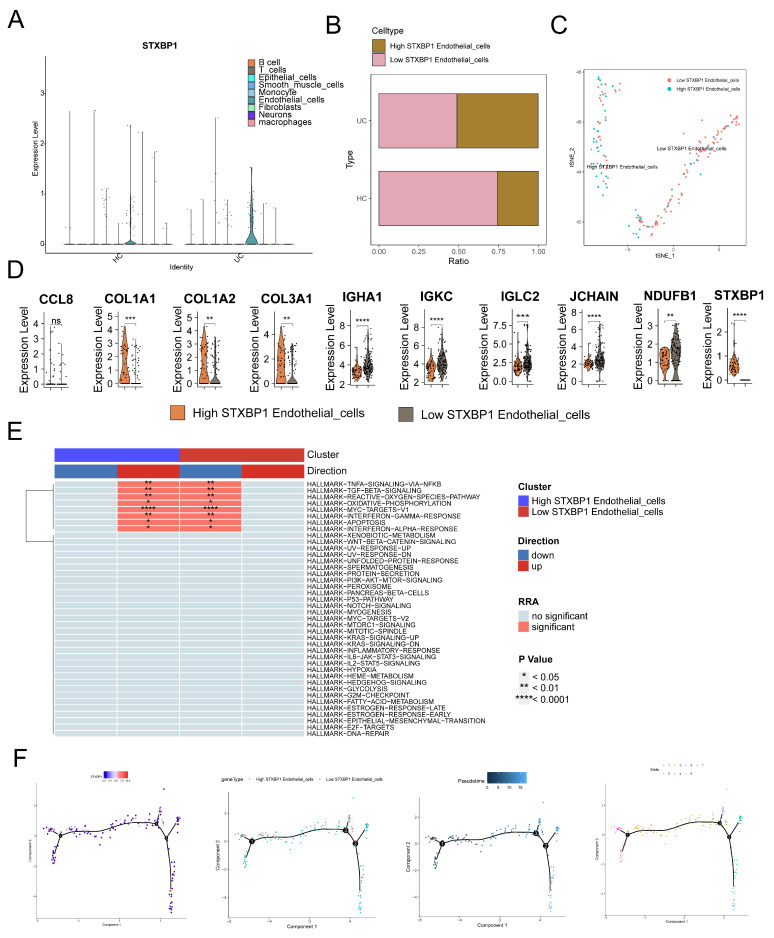
Functional role of STXBP1 in endothelial cells. (**A**) Violin plots showing STXBP1 expression across major cell types. (**B**) Comparison of STXBP1 expression in endothelial cells between UC and HC groups. (**C**) t-SNE plot of endothelial cells colored by STXBP1 expression level (high vs. low, grouped by median expression). (**D**) Violin plots showing the expression levels of differentially expressed genes between STXBP1-high and STXBP1-low endothelial cells. (**E**) Hallmark pathway enrichment analysis in STXBP1-high vs. STXBP1-low endothelial cells. (**F**) Results of cell trajectory and pseudotime analysis of STXBP1 high- and low-expression groups in endothelial cells. ** *p* < 0.01, *** *p* < 0.001, **** *p* < 0.0001; ns, not significant.

We conducted an analysis of STXBP1 expression in various cell types and observed predominant expression in endothelial cells. Furthermore, the expression levels were significantly higher in the UC group compared to the normal group (Figure 11A,B). Subsequently, we employed the t-SNE dimensionality reduction algorithm to classify endothelial cells into high and low STXBP1 expression groups (Figure 11C). We then investigated the differential gene expression patterns between these two groups (Figure 11D). Notably, we observed a significant upregulation of COL1A1, COL1A2, and COL3A1 in the high-expression group relative to the low-expression group.

Additionally, we performed Hallmark pathway enrichment analysis on the endothelial cell subgroups (Figure 11E), revealing heightened activation of inflammatory signaling pathways in the STXBP1 high-expression group. Notably, pathways such as the TNFα/NF-κB signaling pathway, TGFβ signaling pathway, and oxidative phosphorylation displayed increased activity. Furthermore, utilizing trajectory and pseudo time analysis (Figure 11F), we observed a gradual progression of endothelial cells from low STXBP1 expression to high STXBP1 expression over time in both cell groups.

**Figure 12:**
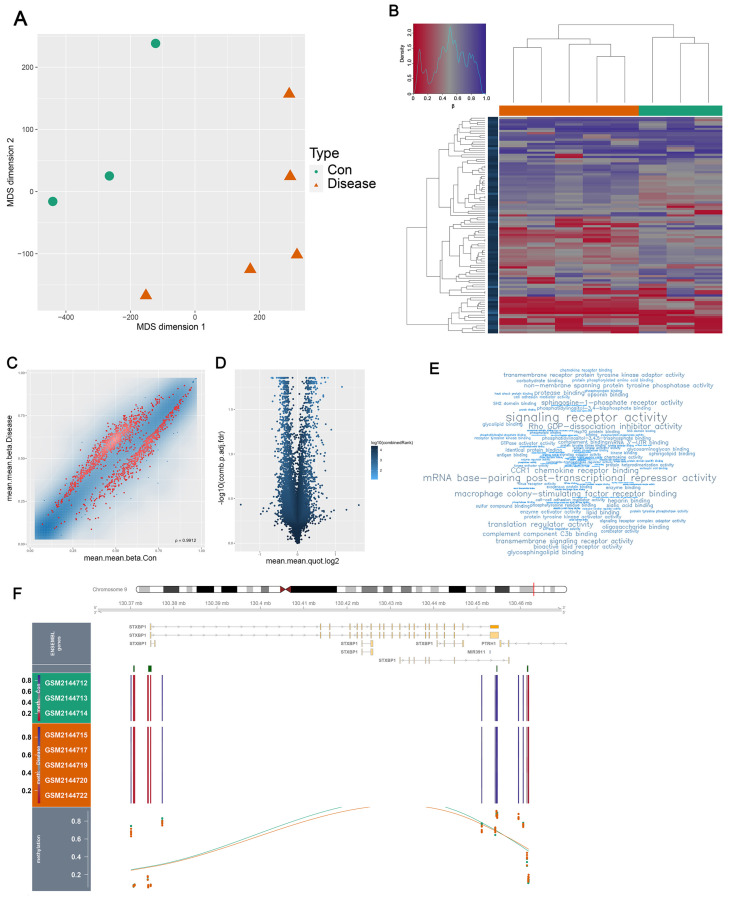
DNA methylation analysis of STXBP1 in UC. Methylation data were obtained from GSE81211. (**A**) PCA plots of the UC group and HC group in the methylation database. (**B**) Heatmap of methylation levels of different genes in the UC group and normal group, each row of the heatmap represents the methylation level of one gene, and each column represents one sample. (**C**) Results of methylation gene differences between UC and HC groups. (**D**) Volcano plots of genes with different methylation levels in the UC group and the normal group. (**E**) Word cloud of enriched methylation genes. (**F**) Gene methylation degree of STXBP1 in the UC group and normal group.

Genes often exhibit correlations with the extent of DNA methylation. To investigate this relationship, we utilized a methylation dataset and conducted a correlation analysis between the UC and HC groups (Figure 12A–E). The heatmap clearly illustrated distinct down- or up-regulation patterns of certain genes, while the volcano map and word cloud from the differential analysis demonstrated the enrichment of relevant genes. Moreover, we specifically examined the degree of methylation of the STXBP1 gene in the UC group compared to the normal group (Figure 12F). Notably, it was evident that the UC group displayed a lower degree of DNA methylation, which consequently led to higher expression levels of the STXBP1 gene within this group.

### 3.8. mRNA Expression Levels of Signature Genes in the Colon of UC Mice

**Figure 13:**
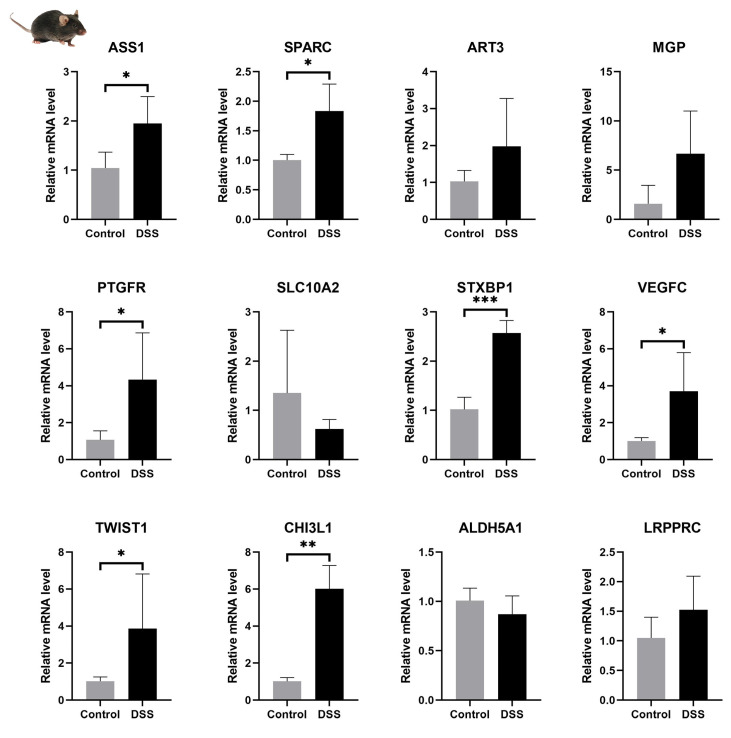
mRNA expression levels of 12 characterized genes in the colon of UC mice. Statistical analysis was performed using Student’s *t*-test: * *p* < 0.05, ** *p* < 0.01 and *** *p* < 0.001.

We constructed a DSS-induced mouse colitis model and extracted total mRNA from mouse colon tissues. mRNA expression levels of 12 characterized genes were then examined. The RT-PCR results are shown in Figure 13 and were largely consistent with our previous analysis. It can be observed that STXBP1 was significantly increased in the DSS group, confirming that STXBP1 plays an important role in UC.

## 4. Discussion

UC is a chronic IBD characterized by inflammation and ulceration, and in some cases, it can progress to colon cancer. Currently, the primary diagnostic approaches for UC rely on endoscopy and histopathological analysis [19]. However, these methods may not be suitable for patients with subtle pathological manifestations. Consequently, there is a need to develop novel diagnostic approaches for UC. Furthermore, achieving mucosal healing is closely associated with the rate of UC remission, and this process often requires long-term treatment [20]. Therefore, it is crucial to explore new therapeutic targets and diagnostic methods to enhance the chances of mucosal healing and improve the overall treatment outcomes for UC patients.

Circadian rhythms exert a profound influence on various biological processes, and numerous studies have established a connection between CRD and a wide range of diseases [21]. This study represents the first attempt to establish a correlation between CRD and UC, aiming to elucidate the intrinsic effects of circadian rhythms on UC and provide a novel approach for its diagnosis, treatment, and prognosis.

Initially, we performed a differential analysis on 86 normal colon tissue samples and 879 UC tissue samples sourced from five datasets. Through this analysis, we identified 162 up-regulated CRD-DEGs and 85 down-regulated CRD-DEGs. Subsequently, in our GSEA, we observed a significant enrichment of genes associated with CRD within the UC group. In addition, our GO, KEGG, and DO enrichment analyses revealed that these CRD-DEGs were predominantly involved in signaling pathways related to cytokine-cytokine receptor interactions, cell chemotaxis, and IL-17 signaling. Notably, these pathways encompass key biological activities such as leukocyte migration, response to LPS, cell differentiation, and immune response. Importantly, these CRD-DEGs demonstrated a clear association with intestinal diseases, including UC.

One of the key characteristics of UC is the presence of chronic inflammation, which involves immune cell infiltration. The transmission pathways of chemokines are closely associated with the recruitment of immune cells, a finding that aligns with our GSEA results. We could posit that CRD contributed to the development of UC by influencing immune cell infiltration. Previous studies [22,23] have demonstrated the intricate connection between immune cell infiltration and UC development, establishing it as a viable diagnostic biomarker for UC. Through an in-depth investigation, we observed a significant correlation between CRD and immune cell infiltration within the UC group. We compared the extent of immune cell infiltration between UC patients and the normal group, as well as among UC patients with varying degrees of circadian dysregulation, revealing a highly consistent pattern.

Intestinal fibrosis is a recognized complication of chronic IBD, and a subset of UC patients also experience the development of intestinal fibrosis. Chiara et al. have conducted a study where they have identified the involvement of smooth muscle cell fibrosis and endothelial-to-mesenchymal cell transition as mechanisms underlying intestinal fibrosis [24]. Interestingly, upon analyzing scRNA-seq data obtained from UC and normal tissues, we observed significant upregulation of CRD-related markers in smooth muscle cells, endothelial cells, and fibroblasts. The crucial role of these cell types in intestinal fibrosis has been further corroborated by another study [25]. This suggests a potential link between CRD and intestinal fibrosis, which warrants further investigation.

We utilized a machine learning algorithm to screen 12 signature genes (ASS1, VEGFC, STXBP1, SPARC, MGP, PTGFR, ART3, TWIST1, CHI3L1, ALDH5A1, LRPPRC, and SLC10A2) from a pool of 247 genes associated with CRD in UC. ASS1 is an enzyme involved in the urea cycle process and is primarily responsible for arginine synthesis in vivo [26]. Previous studies have identified ASS1 as a potential marker of UC and linked it to the infiltration of various immune cells [22]. ASS1 has also been found to play a role in T-cell function, with deficiency leading to primary immune dysfunction [27]. VEGFC is a vascular endothelial growth factor that promotes angiogenesis and the growth of endothelial cells, stimulating their proliferation and migration. It may also have a role in lymphatic vessels. A related study has reported significant protection against the development of acute and chronic colitis in two different animal models with the administration of VEGFC [28].

STXBP1, also known as Munc18-1, is a key regulator of vesicle docking and membrane fusion, playing an essential role in intracellular trafficking and exocytosis [29,30]. Emerging evidence suggests that vesicle trafficking is closely involved in the secretion of inflammatory cytokines and the regulation of immune responses [31]. In addition, neuro-immune interactions have been increasingly recognized as critical regulators of intestinal homeostasis and inflammation [32]. Given the fundamental role of STXBP1 in neurotransmitter release, it is plausible that STXBP1 may influence gut inflammation through modulation of neuro-immune crosstalk. Our single-cell analysis further revealed that STXBP1 is highly expressed in endothelial cells. Endothelial cells are known to play a central role in inflammatory responses by regulating leukocyte recruitment and vascular permeability [33]. Combined with our enrichment analysis showing associations with TNF-α/NF-κB and TGF-β signaling pathways, these findings suggest that STXBP1 may contribute to endothelial dysfunction and inflammatory activation in UC. Moreover, circadian rhythm disruption has been shown to affect immune responses and inflammatory signaling pathways [34]. Therefore, STXBP1 may represent a potential link between circadian dysregulation and intestinal inflammation. However, further experimental studies are required to elucidate the precise molecular mechanisms underlying STXBP1 function in UC.

SPARC is a stromal cell glycoprotein. Gabriela et al. have demonstrated increased gene and protein expression of SPARC in active UC, suggesting it may serve as a marker of intestinal inflammation [35]. SPARC has also been found to promote vascular and multi-organ fibrosis in a TGF-β-dependent manner [36]. MGP is a vitamin K-dependent matrix protein with a high affinity for calcium ions. Its main functions are associated with vascular mineralization and bone tissue. Additionally, MGP has been linked to pulmonary fibrosis [37]. PTGFR is a Prostaglandin F2-α receptor, which has primarily been studied in the context of delivery and its effects on the uterus [38], with no specific studies available on PTGFR in UC. ADP-ribosyltransferase 3 (ART3) has been associated with tumor size [39], but its relationship to UC remains unexplored.

TWIST1 is a transcriptional regulator that can also inhibit the activity of circadian transcriptional activators. TWIST1 is capable of specific autoregulation of Th1 cells, which can mediate the development of enteritis [40,41]. CHI3L1 plays a role in the Th2 inflammatory response and IL-13-induced inflammation, regulating processes such as allergen sensitization, inflammatory cell apoptosis, dendritic cell accumulation, M2 macrophage differentiation, and the invasion of pathogenic intestinal bacteria into the colonic mucosa and lymphoid organs. CHI3L1 interacts with various immune cells and intestinal flora, leading to speculation about its significant role in the pathogenesis of IBD [42]. ALDH5A1 is a succinate-semialdehyde dehydrogenase involved in the degradation of the inhibitory neurotransmitter γ-aminobutyric acid. Studies have shown a positive correlation between the level of ALDH5A1 and tumor progression and metastasis in papillary thyroid carcinoma [43]. LRPPRC plays a role in RNA metabolism within the nucleus and mitochondria. It has been identified as a key functional downstream factor and therapeutic target in P53 mutation-induced chemoresistance in colorectal cancer [44].

SLC10A2 is an Ileal sodium/bile acid cotransporter that plays a crucial role in the sodium-dependent reabsorption of bile acids in the lumen of the small intestine.

By developing a nomogram based on these 12 genes, we created new models for the prediction and early diagnosis of UC. Our analysis demonstrated a strong correlation between these 12 genes and immune cells. Notably, TWIST1, SPARC, PTGFR, MGP, and CHI3L1 exhibited a robust positive correlation with neutrophils and a significant negative correlation with M2 macrophages. This finding suggested that the expression of these signature genes was closely intertwined with the development of inflammation in UC. Furthermore, in the analysis of other cell types, we observed varying degrees of aberrant expression of these genes in smooth muscle cells, endothelial cells, and fibroblasts. Importantly, these 12 signature genes were also associated with intestinal fibrosis. The mRNA expressions of these 12 characterized genes in UC mouse colon tissues were also substantially consistent with our analysis.

Through consensus clustering analysis, we identified two distinct subtypes of UC characterized by CRD. The differences between these subtypes primarily manifested in inflammation-related signaling pathways. Notably, Cluster 2 exhibited higher levels of inflammation compared to Cluster 1; however, the Toll-like receptor transduction pathway, chemokine signaling pathway, and NOD-like receptor signaling pathway were significantly downregulated in Cluster 2 compared to Cluster 1. This finding suggested that a comprehensive investigation into subtypes and diagnostic markers of UC CRD could enhance our understanding of UC pathogenesis and offer insights for individualized diagnosis, treatment, and prognosis of patients with different subtypes.

Although the nomogram constructed in this study demonstrated high predictive accuracy in independent transcriptomic datasets, it is based solely on public gene expression data and therefore represents a computational model rather than a clinically validated tool. Further validation in prospective clinical cohorts is required before clinical application.

In our present analysis, we observed that STXBP1 emerged as the shared signature gene among all three algorithms, as depicted in the Venn diagram. Notably, the STXBP1 high-expression group exhibited significant enrichment of inflammation-related pathways. Through GSVA, we found that the samples in this group demonstrated reduced adhesion of smooth muscle cell–matrix, negative effects on vascular endothelial cells, inhibition of cytokine and cell adhesion molecule signaling, as well as enhanced metabolic processes of certain amino acids and salts. Moreover, within endothelial cells, the STXBP1 high-expression group exhibited a stronger inflammatory signaling pathway. These findings suggested that STXBP1 expression had the potential to influence immune responses and the progression of intestinal fibrosis.

## 5. Conclusions

This study represented the first exploration of the involvement of CRD in the pathogenesis of UC. We conducted a comprehensive analysis using various bioinformatics methods to elucidate the intrinsic relationship between CRD and UC. Previous differential gene screening and enrichment analysis showed that genes associated with CRD are associated with immune responses and immune cells in UC. The immune cell infiltration analysis showed a strong correlation between CRD and the degree of immune cell infiltration in UC patients. We also used machine learning algorithms to analyze 12 signature genes associated with CRD in UC for in-depth analysis of the relationship between CRD and UC. By examining the expression patterns of the 12 signature genes across different dimensions, we developed a novel nomogram and identified distinct subtypes of UC. The nomogram demonstrated good predictive performance for UC risk. Collectively, these findings offered novel insights and methodologies for the early diagnosis, treatment, and prognosis of UC.

## Figures and Tables

**Table 1:** Basic information about the bulk and single cell RNA-seq data set for ulcerative colitis.

GEO Dataset	Samples (UC/Normal)	Platform	Tissue Source	Dataset Description	Normalization Method	Data Type	Set Type
GSE87466	87/21	GPL13158	Colon	Active UC and HC colonic mucosal biopsies	RMA	Bulk RNA-seq	Training
GSE75214	97/11	GPL6244	Colon	Active UC and HC colonic mucosal biopsies	RMA	Bulk RNA-seq	Training
GSE206285	550/18	GPL13158	Colon	Active UC and HC colonic mucosal biopsies	RMA	Bulk RNA-seq	Training
GSE87473	106/21	GPL13158	Colon	Active UC and HC colonic mucosal biopsies	RMA	Bulk RNA-seq	Training
GSE47908	39/15	GPL570	Colon	Active UC and HC colonic mucosal biopsies	RMA	Bulk RNA-seq	Training
GSE16879	24/12	GPL570	Colon	Active UC and HC colonic mucosal biopsies	RMA	Bulk RNA-seq	Validation
GSE48958	13/8	GPL6244	Colon	Active UC and HC colonic mucosal biopsies	RMA	Bulk RNA-seq	Validation
GSE53306	28/12	GPL14951	Colon	Active UC and HC colonic mucosal biopsies	Quantile	Bulk RNA-seq	Validation
GSE38713	30/13	GPL570	Colon	Active UC and HC colonic mucosal biopsies	RMA	Bulk RNA-seq	Validation
GSE231993	4/4	GPL1857	Colon	Single-cell RNA-seq of UC and HC	SCTransform	scRNA-seq	Other Data
GSE81211	8/3	GPL13534	Colon	DNA Methylation profiles of UC and HC	SWAN/Beta-mixture	Methylation	Other Data

**Table 2:** Primers used for real-time PCR.

Gene	RT-PCR Primer Sequence (5′ to 3′)
m-β-Actin	Forward sequence ATGTGGATCAGCAAGCAGGAG
	Reverse sequence GGTGTAAAACGCAGCTCAGTAAC
m-ASS1	Forward sequence ACACCTCCTGCATCCTCGT
	Reverse sequence GCTCACATCCTCAATGAACACCT
m-SPARC	Forward sequence GGCCCGAGACTTTGAGAAGA
	Reverse sequence AATGTTCCATGGGGATGAGG
m-ART3	Forward sequence TCTGGCAATACGCTCCTTCC
	Reverse sequence GTGGGATCCCCATAGCAGTC
m-MGP	Forward sequence AGAGAGTCCAGGAACGCAAC
	Reverse sequence CGGTTGTAGGCAGCGTTGTA
m-PTGFR	Forward sequence TAATGTGCGTCTCCTGCGTC
	Reverse sequence GCCATGCGGAGAGCAAAAAG
m-SLC10A2	Forward sequence GTGGGCTTCCTCTGTCAGTT
	Reverse sequence CCAGGGCAGCAACCCATAA
m-STXBP1	Forward sequence CGGTCCCCGCCTCATTATTT
	Reverse sequence TGCGTGGATCCTATCAGCAC
m-VEGFC	Forward sequence GCTTTTGAAGGCAAAGACCTGG
	Reverse sequence AGTCTGGGTACAGGACAGACAT
m-TWIST1	Forward sequence CCACCCCACTTTTTGACGAAG
	Reverse sequence GTCAGTGGCTGATTGGCAAG
m-CHI3L1	Forward sequence TGGATCTCGCCTGGCTCTAC
	Reverse sequence CTGCGCTGAGCAGGAGTTTC
m-ALDH5A1	Forward sequence GAGATCCTGCAGCTCGCTT
	Reverse sequence GAAGCTCCGCAGCAGAAAG
m-LRPPRC	Forward sequence TCCAGGGTATCCCGCGAA
	Reverse sequence CGCCCCTAGTGGGACAAAC

**Table 3:** Performance comparison of three machine learning algorithms on the validation set.

Algorithm	Number of Features	AUC (95% CI)	Accuracy	Sensitivity (Recall)	Specificity	F1-Score
LASSO	62	1.000 (1.000–1.000)	0.985	1	0.97	0.985
Random Forest	20	0.987 (0.973–0.996)	0.942	0.935	0.948	0.941
SVM-RFE	12	0.994 (0.987–0.999)	0.968	0.962	0.974	0.968

## Data Availability

All data generated or analyzed during this study are included in this published article.

## Supplementary Materials

The following supporting information can be downloaded at: https://www.mdpi.com/article/10.3390/genes17040383/s1, Figure S1: (A,B) Box plots of 5 gene sets (GSE206285, GSE47908, GSE75214, GSE87466 and GSE87473) before and after correction. (C) Scatter plot of the correlation between CRD-DEGs and immune cell infiltration; Figure S2: ROC curves of predicted genes from LASSO and RF.
