# Supplementary figures and images for: Unraveling Circadian Rhythm Disorder-Related Gene Signatures and Molecular Subtypes in Ulcerative Colitis: An Analysis of Bulk and Single-Cell Transcriptomics
Source: Genes (Basel). 2026 Mar 27;17(4):383. doi: 10.3390/genes17040383 (PMC13115594; doi:10.3390/genes17040383)

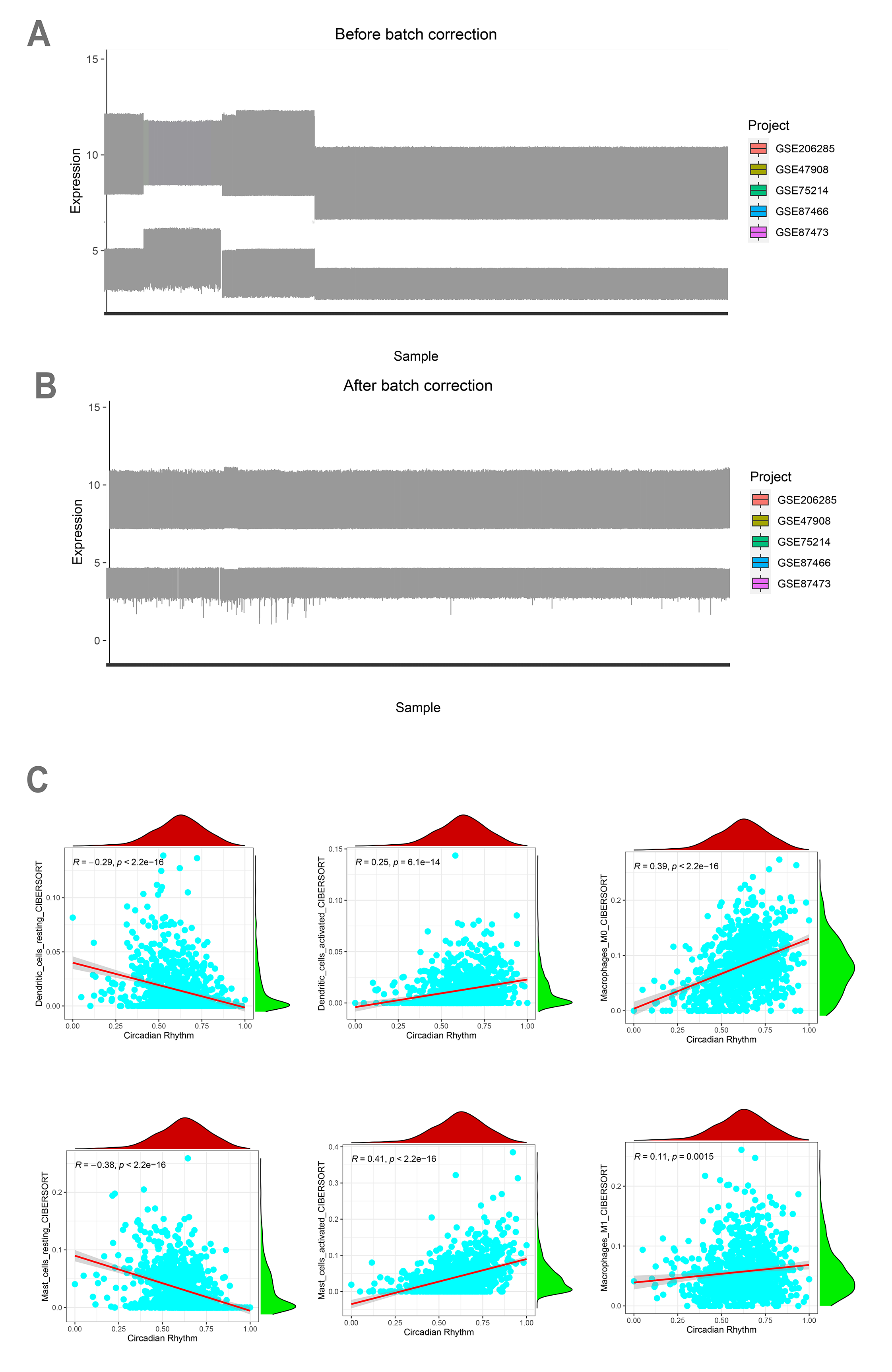

Supplement: Supplementary file 1 [file genes-17-00383-s001.zip › Figure S1.tif]

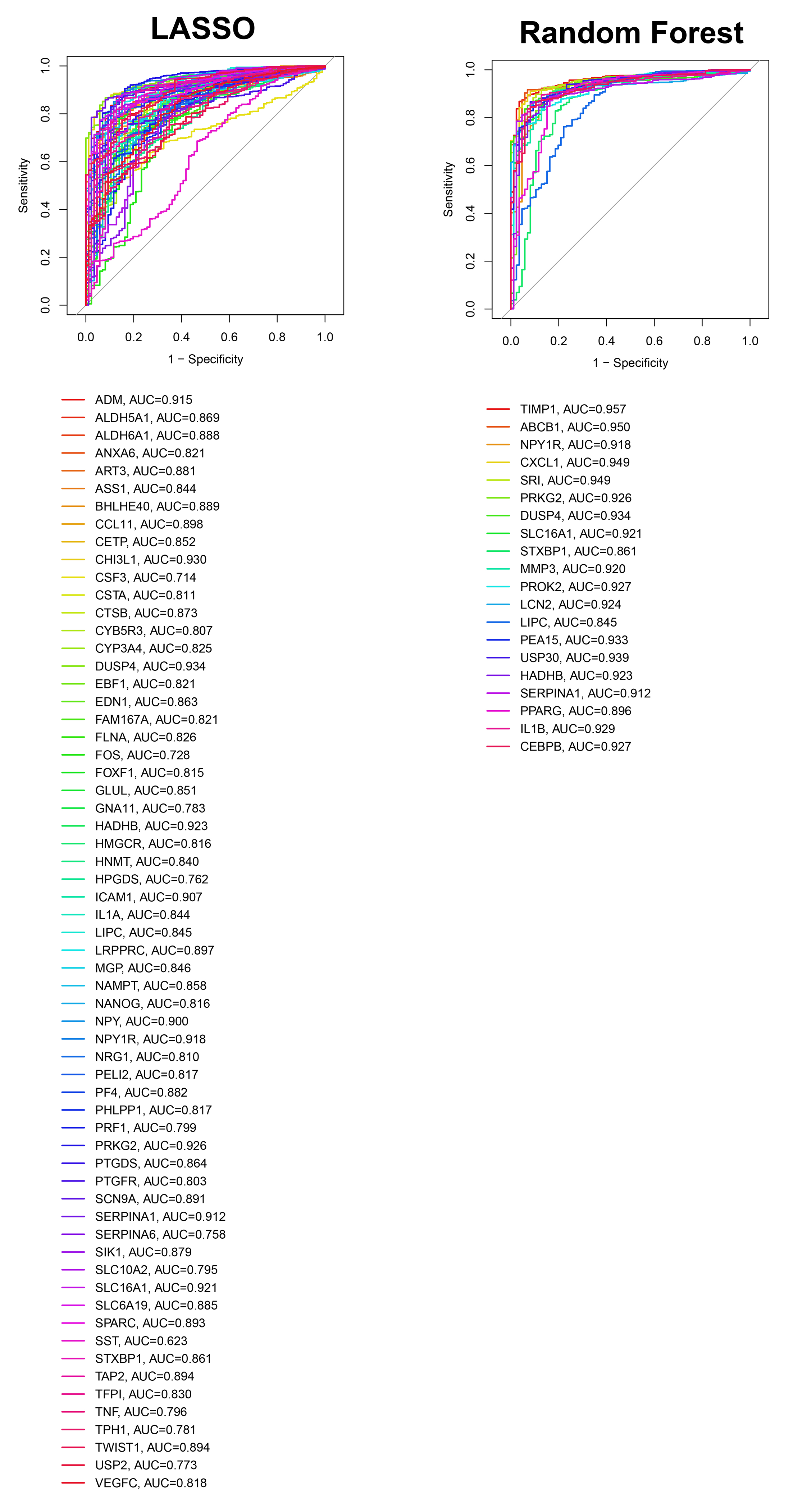

Supplement: Supplementary file 1 [file genes-17-00383-s001.zip › Figure S2.tif]
